# Risk prediction of cholangitis after stent implantation based on machine learning

**DOI:** 10.1038/s41598-024-64734-w

**Published:** 2024-06-14

**Authors:** Rui Zhao, Lin Gu, Xiquan Ke, Xiaojing Deng, Dapeng Li, Zhenzeng Ma, Qizhi Wang, Hailun Zheng, Yong Yang

**Affiliations:** 1 The First Affiliated Hospital of Bengbu Medical University, Yanhuai Road, Bengbu, 233000 China; 2 School of Mechanical Engineering, Hefei University of Technology, Hefei, 230009 China

**Keywords:** Malignant obstructive jaundice, Endoscopic retrograde cholangiopancreatography, Cholangitis, Machine learning model, Hepatology, Biliary tract, Biliary tract disease

## Abstract

The risk of cholangitis after ERCP implantation in malignant obstructive jaundice patients remains unknown. To develop models based on artificial intelligence methods to predict cholangitis risk more accurately, according to patients after stent implantation in patients’ MOJ clinical data. This retrospective study included 218 patients with MOJ undergoing ERCP surgery. A total of 27 clinical variables were collected as input variables. Seven models (including univariate analysis and six machine learning models) were trained and tested for classified prediction. The model’ performance was measured by AUROC. The RFT model demonstrated excellent performances with accuracies up to 0.86 and AUROC up to 0.87. Feature selection in RF and SHAP was similar, and the choice of the best variable subset produced a high performance with an AUROC up to 0.89. We have developed a hybrid machine learning model with better predictive performance than traditional LR prediction models, as well as other machine learning models for cholangitis based on simple clinical data. The model can assist doctors in clinical diagnosis, adopt reasonable treatment plans, and improve the survival rate of patients.

## Introduction

Malignant obstructive jaundice (MOJ) refers to a disease in which the bile duct, liver, gallbladder, pancreas, or periampullary of the duodenum are invaded by primary tumors or metastases from other sites, and the obstruction or compression of the bile duct obstructs bile drainage and causes corresponding clinical symptoms^1^. MOJ has an insidious onset and rapid growth. When clinical symptoms appeared, it was often indicated that the tumor had advanced, and most patients had no chance to remove the malignant tumor^2^.

Currently, endoscopic retrograde cholangiopancreatography (ERCP) biliary stent implantation has become the first choice for MOJ patients with no chance of radical surgical treatment. However, ERCP was still an invasive operation. Due to the reasons of the lesion itself and operation, postoperative biliary system infection was easy to occur, including acute cholangitis, acute cholecystitis, liver abscess, etc., among which cholangitis was the most common, if not timely intervention, can aggravate the disease progression^3^. Especially after endoscopic sphincterotomy (EST) or column balloon expansion, intestinal flora can also invade the blood through the damaged epithelium and cause bacteremia or even systemic infection^4,5^. If not treated in time, it was easy to cause systemic inflammatory response syndrome (SIRS), septic shock and even death, and the case fatality rate often exceeds 10%, even up to 27%^6^. Biliary infection was a serious and life-threatening infectious disease. Studies have shown that the incidence of early cholangitis after ERCP is 0.28–27.80%^7–9^, and according to the 2013 Tokyo guideline, the fatality rate of acute cholangitis has been reported to range from 2.7 to 10%^10^. If not well controlled, cholangitis can lead to sepsis, which can further seriously endanger the patient’s life. However, predicting the risk of cholangitis after ERCP and finding clinically relevant risk factors were significant for postoperative recovery.

Traditional statistical methods can be used to find several risk factors related to the disease and build prediction models, such as linear regression, logistic regression, Cox regression, etc. Such predictions were found to be significantly more accurate than empirical predictions^11,12^. With the continuous progress of computer technology, medical diagnosis technology based on artificial intelligence (AI) developed rapidly. Machine learning (ML) technology can analyze databases and has considerable advantages in screening, assimilation, evaluating massive and complex medical data. ML technology has been used in the auxiliary diagnosis and treatment of various diseases, including biliary diseases^13–16^, hepatocellular carcinoma^17–19^, and biliary cholangitis^20,21^. However, few studies have used ML models to predict the risk of cholangitis after ERCP.

In this work, a hybrid machine learning model based on beetle antennae search (BAS) algorithm and random forest (RF) model was designed to predict the risk of cholangitis in patients after ERCP, thereby helping to identify potential patients who may develop cholangitis, and compare it with a traditional logistic regression (LR) prediction model and other ML model.

## Material and methods

### Patients

The First Affiliated Hospital of Bengbu Medical College conducted the current single-center retrospective investigation (Bengbu, China). The electronic ERCP database was used to acquire the demographic information and specifics of the ERCP surgery for MOJ patients who underwent ERCP between January 2017 and October 2022. The following requirements were fulfilled by the participants in this study: age requirement of 18 years or older; MOJ diagnosis on clinical or histopathologic examination. Patients were excluded from this study for the following reasons: (a) acute cholangitis occurred prior to ERCP; (b) organ failure was present prior to ERCP; (c) any surgery was completed within one month prior to ERCP; (d) death following ERCP from a cause other than acute cholangitis; and (e) clinical data were lacking.

To determine the initial diagnosis and ERCP indications, all patients with complete information from hospital records on general data, laboratory testing, and imaging examination were included, and contraindications were excluded. The Ethics Committee of the First Affiliated Hospital of Bengbu Medical College authorized this investigation (Bengbu, China; approval NO.2019KY030).

### Surgical procedure

All ERCP procedures were carried out or overseen by licensed doctors with at least ten years of ERCP experience. Slowly insert the duodenal endoscope (OlympusJF-260/TJF-260) through the mouth, enter the descending duodenal segment, clarify the duodenal papilla, straighten the short mirror, and place the contrast catheter into the endoscopic clamp placement tube hole. Properly adjust the angle button and clamp lifter so that the catheter was perpendicular to the open end of the papilla and placed inside the papilla into the common bile duct or pancreatic duct. After the guide wire is positioned at the lesion, 10–20 ml of Iohexol Injection (GE HEALTHCARE (Shanghai) Co., Ltd., specification: 50 ml) was injected for imaging, and the lesion was observed under X-ray. Depending on the patient's lesion, different endoscopic treatment measures were selected, and bile duct plastic or metal stents were used for internal drainage.

### Primary study endpoints

The study cut-off date was December 2022, and the follow-up endpoint was 30 days postoperatively, with or without cholangitis. For the definition of cholangitis, refer to the Tokyo Guidelines 2018^22^ and European Society of Gastrointestinal Endoscopy (ESGE) Guideline^23^: after 24 h after the end of the operation, the presence of a temperature ≥ 38 °C and/or a white blood cell count (WBC) > 10.0 × 10^9^/L, elevated biochemical indicators such as serum bilirubin and biliary enzymes reflecting cholestasis, and imaging changes related to the etiology of cholestasis.

### Clinical dataset

The following information was collected and recorded: the patient’s personal information (i.e., gender, age, body mass index, prior medical conditions, and prior surgical history), tumor-related indicators (i.e., tumor type, obstruction length, obstruction location, stent type (a single plastic stent or a single metal stent), and duration of obstruction), and blood routine indices before ERCP (i.e., white blood cell count, total bilirubin, direct bilirubin, albumin, and blood glucose), and used antibiotic before ERCP and time of operation. A detailed description of a heterogeneous dataset comprising 16 quantitative features and 11 qualitative features (n = 16 + 11 = 27) was shown in Table 1. Finally, the study enrolled 218 patients. The data set was separated into a training set (70%) and a test set (30%) to evaluate the model's accuracy.

**Table 1:** General characteristics of the study population.

Features	Cholangitis (n = 49)	No-cholangitis (n = 169)	Statistics	*P* value
Gender, n (%)			Χ^2^ = 1.541	0.214
Male	31 (63.27)	90 (53.25)		
Female	18 (36.73)	79 (46.75)		
Age (years), mean (SD)	67.61 ± 11.77	70.52 ± 12.61	t = 1.498	0.851
BMI (kg/m^2^)	21.25 ± 2.86	21.14 ± 3.39	t = 0.233	0.816
Child–Pugh grade, n (%)			Χ^2^ = 1.483	0.223
A	30 (61.22)	119 (70.41)		
B	19 (38.78)	50 (29.59)		
Diabetes mellitus, n (%)			Χ^2^ = 0.277	0.599
Yes	5 (10.20)	22 (13.02)		
No	44 (89.80)	147 (86.98)		
Cholecystolithiasis, n (%)			Χ^2^ = 3.516	0.061
Yes	6 (12.24)	42 (24.85)		
No	43 (87.76)	127 (75.15)		
Obstruction position, n (%)			Χ^2^ = 20.653	< 0.010
High	14 (28.57)	110 (65.09)		
Low	35 (71.43)	59 (34.91)		
Abdominal surgery, n (%)			Χ^2^ = 1.572	0.210
Yes	6 (12.24)	34 (20.12)		
No	43 (87.76)	135 (79.88)		
PTCD, n (%)			Χ^2^ = 2.708	0.100
Yes	8 (16.33)	14 (8.28)		
No	41 (83.67)	155 (91.72)		
ERCP, n (%)			Χ^2^ = 0.399	0.528
Yes	2 (4.08)	11 (6.51)		
No	47 (95.92)	158 (93.49)		
Chemo therapy, n (%)			Χ^2^ = 0.147	0.702
Yes	7 (16.57)	28 (16.57)		
No	42 (83.43)	141 (83.43)		
Stent type, n (%)			Χ^2^ = 2.469	0.117
Plastic stent	32 (65.30)	89 (52.67)		
Metal stent	17 (34.70)	80 (47.33)		
Tumor type, n (%)			Χ^2^ = 16.092	0.030
Cholangiocarcinoma	32 (65.31)	69 (40.83)		
Pancreatic cancer	4 (8.16)	58 (34.32)		
Duodenal papillary carcinoma	2 (4.08)	15 (8.88)		
Ampulary carcinoma	3 (6.12)	7 (4.14)		
Metastasis cancer	8 (16.33)	20 (11.83)		
Length of obstruction (cm), mean (SD)	3.22 ± 1.29	2.83 ± 1.08	t = 2.170	0.031
WBC (× 10^9^/L), mean (SD)	7.78 ± 5.17	7.09 ± 4.24	t = 0.858	0.394
Hb (g/L), mean (SD)	116.39 ± 19.97	113.53 ± 19.16	t = 0.889	0.377
TBIL (μmol/L), mean (SD)	216.89 ± 131.07	172.58 ± 107.86	t = 2.163	0.034
DBIL (μmol/L), mean (SD)	163.99 ± 112.10	145.93 ± 97.37	t = 2.153	0.035
Alb (g/L), mean (SD)	34.81 ± 6.95	34.27 ± 5.21	t = 0.508	0.613
Glu (mmol/L), mean (SD)	5.93 ± 2.19	6.02 ± 2.66	t = 0.230	0.816
Obstruction of time (day), mean (SD)	24.86 ± 33.07	25.47 ± 37.17	t = 0.111	0.912
Operation of time (min), mean (SD)	46.71 ± 7.07	38.63 ± 5.67	t = 7.343	< 0.010
Used antibiotics before ERCP, n (%)			Χ^2^ = 1.157	0.282
Yes	15 (30.61)	39 (23.08)		
No	34 (69.32)	130 (76.92)		

### Univariant statistical analysis

Univariant statistical analysis were performed using Python software (version 3.8.8). For normally distributed data, continuous variables were expressed as mean ± standard deviation (SD), and comparison was performed by *t* test and these categorical variables examined by Pearson’s chi-squared test. Nonparametric tests would be used to compare other continuous variables, which were expressed as the median and interquartile range (IQR). The performance of each variable was compared by the area under the receiver operating characteristic curve (AUROC).

### Model development and optimization

We applied 6 different ML classification methods: random forest (RF), logistic regression (LR), support vector machines (SVM), decision tree (DT), Naive Bayes (NB) with Gaussian class-conditional density functions, and Adaboost (AB). Tenfold Cross-validation was used throughout the experiment. AUROC was selected as the primary evaluation index. The parameter tuning of RF model was completed by the BAS algorithm. Additional details on hyperparameter setting and BAS algorithm were provided in the Supplementary material.

In order to make the ML model work better, we assessed the variables that were clinically significant and calculated their weight in influencing the clinical outcome. To that end, Shapley additive explanations (SHAP) and RF models were applied to find clinical correlations of different variables.

The following experiment was an extension of the above experiment. Features were selected according to the evaluation of the relative importance of features by the RF model, and then the hyperparameters were adjusted. In this experiment, this choice will be made by a model. Table S2 presents in detail the parameters of the experiment. These parameters include the RF model parameters and features. 0 indicated a feature was deleted, and 1 indicated a feature was reserved. The deletion of features started with less relevant features. AUROC of the test set was taken as the fitness value. Fig. S1 shows the experiment schematics.

### Metrics

The standard indicators, accuracy (ACC), F1-score, mean squared error (MSE) and area under the receiver operating characteristic curve (AUROC) values were selected to evaluate the model' performance^24^. These indicators were used to show the final results of the classification. Models were implemented using Python 3.8.8. The Python language, Pandas, NumPy, and Sklearn packages were used in this work.

### Ethics approval and consent to participate

The study protocol complied with the standards of the Declaration of Helsinki and obtained approval from the Institutional Review Board of the First Affiliated Hospital of Bengbu Medical College (Bengbu, China; approval NO.2019KY030).

The Ethical Committee of Clinical Medical Research of the First Affiliated Hospital of Bengbu Medical College waived the need to obtain informed consent from study participants.

## Results

### Characteristics of patients

Table 1 shows the distributions of baseline characteristics of patients. The 2 groups significantly differed in the distributions of obstruction position (OP), tumor type (TT), length of obstruction (LO), total bilirubin (TBIL), direct bilirubin (DBIL), and operation of time (OT) (*P* < 0.05). On average, patients with cholangitis were less likely to be female (36.73% vs. 63.27%). Compared with patients with no-cholangitis, patients with cholangitis had higher average length of obstruction (LO), white blood cell (WBC), hemoglobin (HB), TBIL, DBIL, and operation of time (OT).

**Figure 1:**
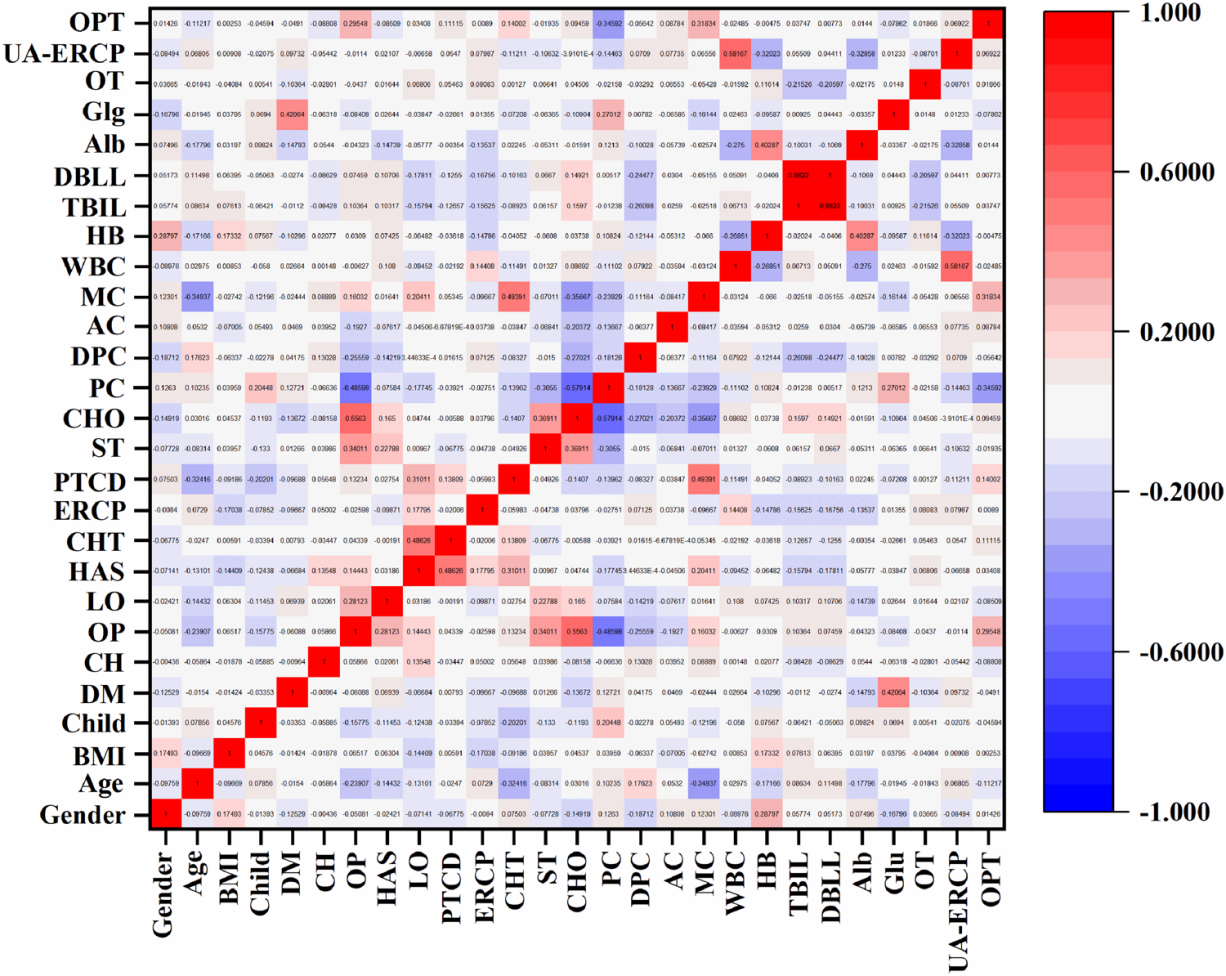
Correlation between features.

One step in data preprocessing was to check the collinearity of features in the dataset. The model will be unstable if there are two collinearity strongly correlated features. Figure 1 shows the correlation coefficients between the calculated feature pairs. It can be seen that the correlation coefficients of the 3 feature pairs are more significant than 0.5. DBIL was strongly positively correlated with TBIL and used antibiotics before ERCP was positively correlated with WBC, while Obstruction position was weakly correlated with pancreatic cancer. The correlation coefficient of a feature pair is less than − 0.5. Cholangiocarcinoma and Pancreatic cancer were negatively correlated. All the features were relatively independent and could better describe the characteristics of variables when they were used as model inputs.

### Univariant statistical analysis

**Figure 2:**
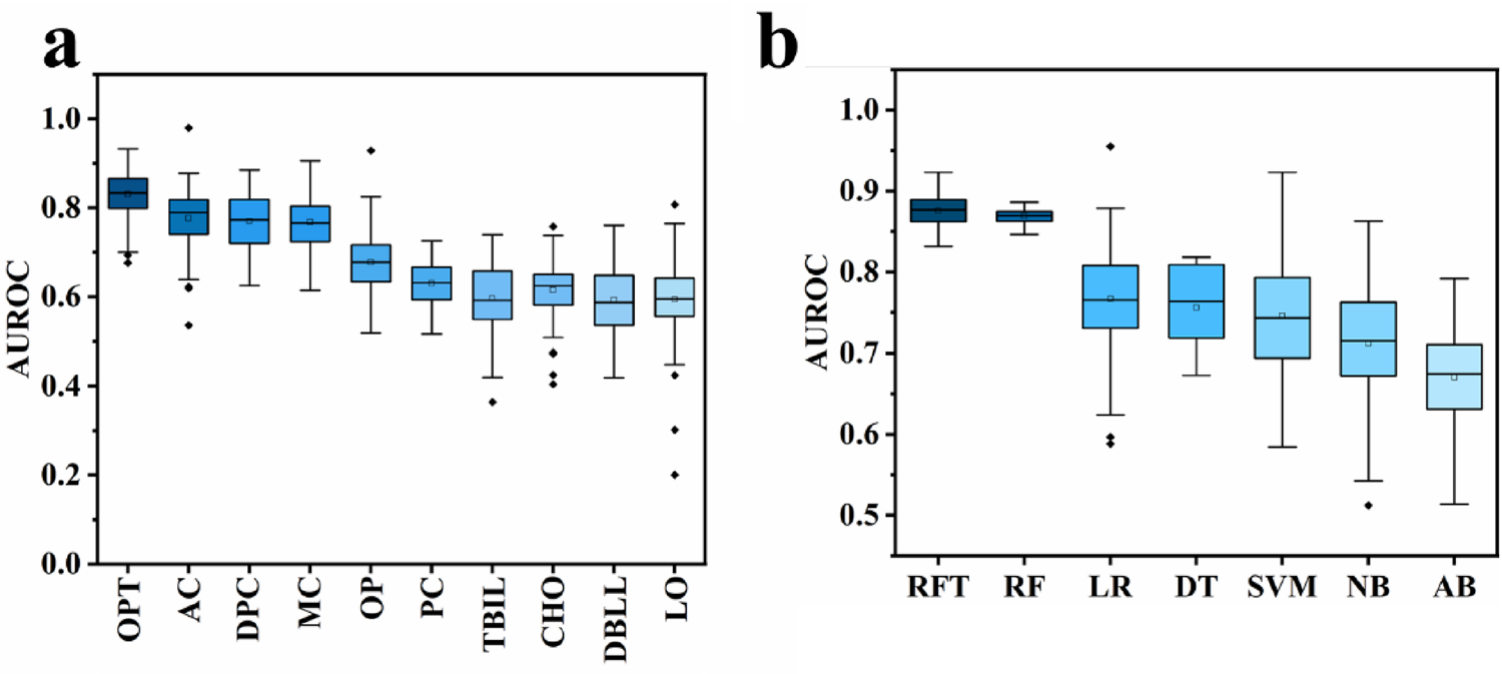
(**a**) Comparison of performance of univariate statistical analysis in AUROC, (**b**) comparison of performance of each machine learning model in AUROC.

We performed a univariate statistical analysis for statistically significant features. As shown in Fig. 2a and Table 2, operation of time had the highest AUROC (0.83), and length of obstruction had the lowest performance with AUROC of 0.59. Among the four statistically different tumor type variables, ampulla cancer had the best AUROC of 0.78, but it was not significantly higher than duodenal papillary and metastatic cancers. The prediction performance of pancreatic cancer and cholangiocarcinoma was slightly worse.

**Table 2:** Predict results for univariant statistical analysis.

Feature	Accuracy-training	Accuracy-testing	AUROC-training	AUROC-testing
Operation of time	0.79	0.80	0.82	0.83
Ampulla carcinoma	0.84	0.80	0.89	0.78
Duodenal papillary carcinoma	0.85	0.80	0.89	0.77
Metastatic cancer	0.85	0.80	0.89	0.77
Obstruction position	0.77	0.78	0.68	0.68
Pancreatic cancer	0.78	0.77	0.63	0.63
TBIL	0.77	0.78	0.59	0.60
Cholangiocarcinoma	0.78	0.77	0.63	0.61
DBIL	0.77	0.78	0.60	0.59
Length of obstruction	0.78	0.78	0.59	0.59

### Machine learning classifiers

The performance of each ML model is listed in Fig. 2b and Table 3. The RF model with optimized parameters by the BAS algorithm was used to predict the risk of Cholangitis. BAS algorithm can accelerate the search speed and avoid the search process falling into local optimal. The accuracy and AUROC of the training set in RFT, RF, DT, and AB have achieved the ideal state (accuracy: 1.00). Performance on the test set has declined. RFT had the highest AUROC (0.87). Other models overall performed poorly, with lower accuracies. AB had the lowest AUROC (0.67) and the lowest accuracy (0.71).

**Table 3:** Predict results for each machine learning model.

Methods	Accuracy-training	Accuracy-testing	F1-score-training	F1-score-testing	AUROC-training	AUROC-testing	MSE-training	MSE-testing
RFT	0.99	0.86	0.99	0.57	1.00	0.87	0.0065	0.1364
RF	0.99	0.85	0.99	0.5	1.00	0.86	0.0066	0.1515
LR	0.84	0.85	0.47	0.5	0.86	0.82	0.1645	0.1515
DT	1.00	0.83	1.00	0.62	1.00	0.78	0.0	0.1667
SVM	0.83	0.83	0.43	0.27	0.97	0.75	0.1711	0.1667
BN	0.81	0.76	0.43	0.52	0.78	0.72	0.1908	0.2424
AB	1.00	0.71	1.00	0.34	1.00	0.67	0.0	0.2879

### Feature selection

**Figure 3:**
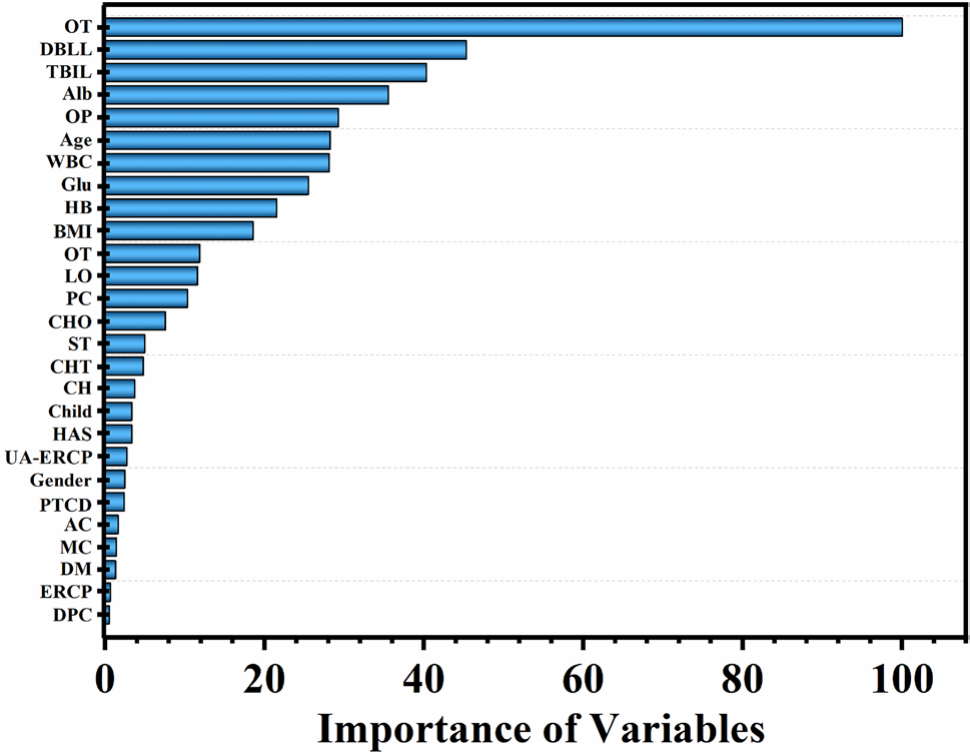
Variable importance of features in RFT model.

**Figure 4:**
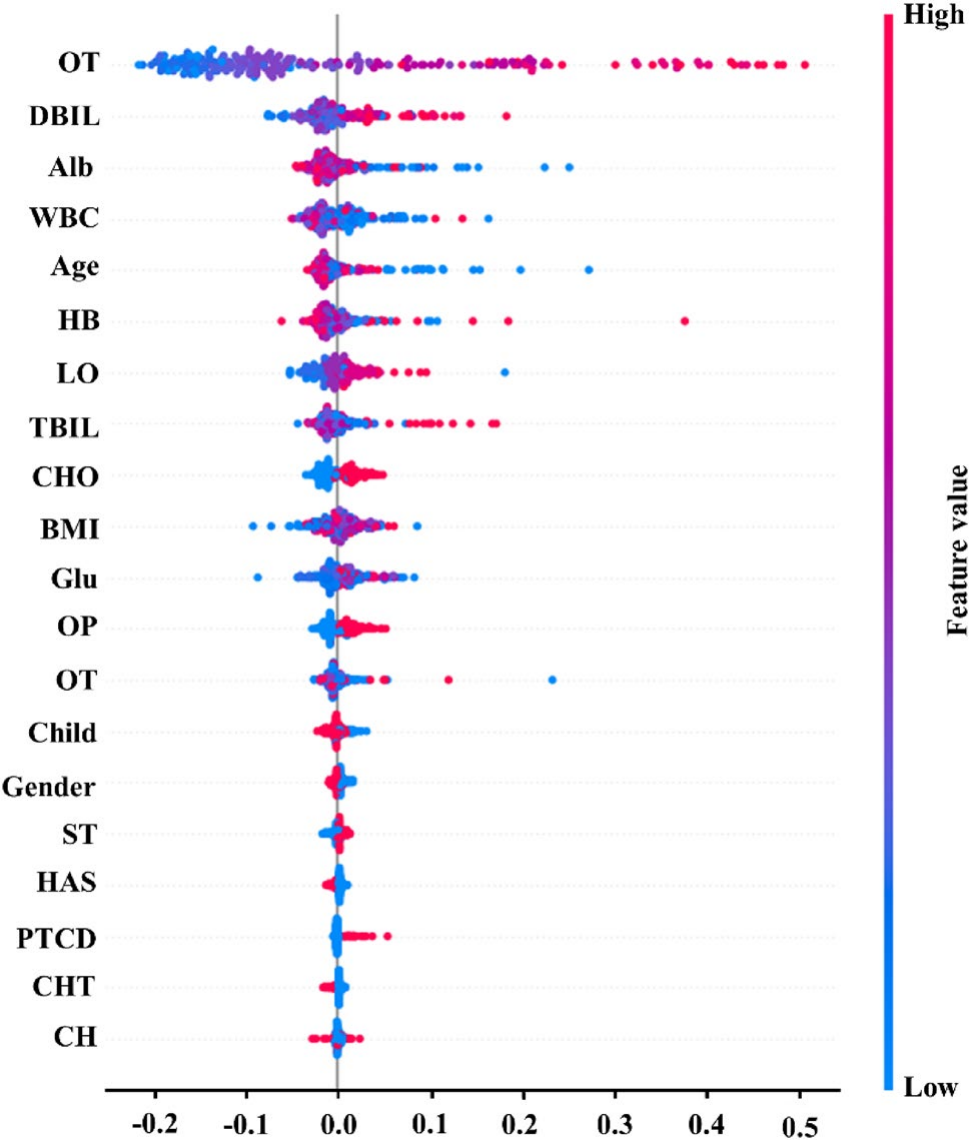
SHAP value on selected feature.

Figure 3 shows the predictors by their relative importance in the RF model after tuning the parameters, with high-importance values representing the most influential variables for predicting the risk of cholangitis. These features have high predictive value, including operation of time, DBLL, TBIL, albumin, and obstruction position. In addition, and as shown by the descriptive analyses from the random forests, other features were identified as having a potential but weaker influence, including duodenal papillary carcinoma, ERCP et al. After that, we further examined the clinical relevance of different variables in RFT model on the feature set with selected variables using the SHAP method (Fig. 4). The results of feature importance were similar to the results of feature importance analysis based on RFT model and statistically significant features (*P* < 0.05).

**Figure 5:**
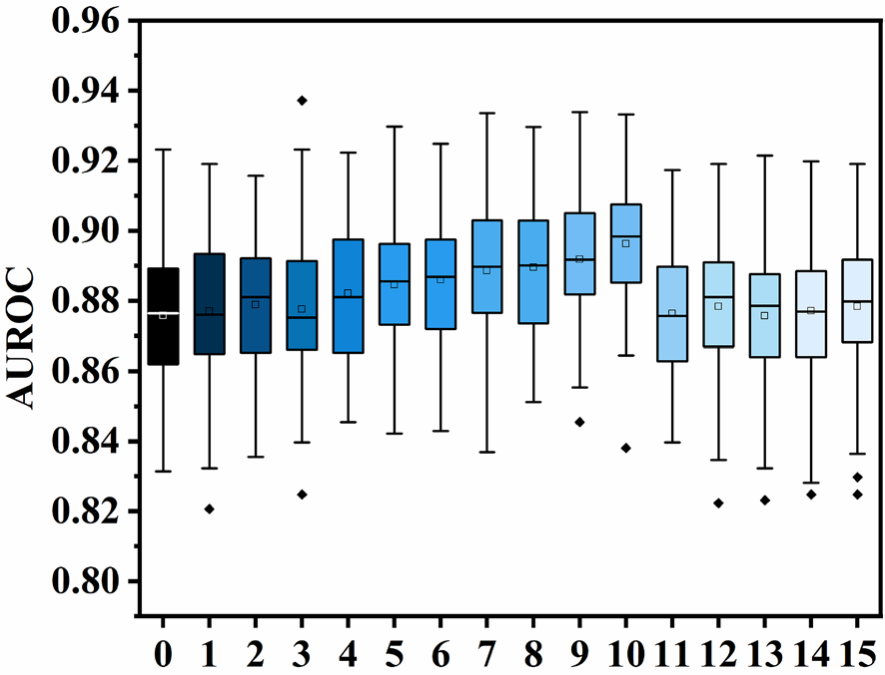
Comparison of performance of different selection features in AUROC.

The BAS algorithm used the RF model with optimized parameters and features to predict the risk of cholangitis. The model has the highest prediction accuracy in the test set after deleting six-ten features with the weakest prediction performance from the data set (Fig. 5). The accuracy of the training set has achieved the ideal state (AUROC: 1.00). They were using the test set AUROC of 0.89. The prediction accuracy of the model was improved by feature selection.

## Discussion

ERCP biliary stenting was the preferred and effective palliative conservative treatment for MOJ with no chance of radical surgery^25^. Biliary infection, especially cholangitis, was a common complication after ERCP and can lead to death due to septic shock in severe cases^26^. The incidence of cholangitis after ERCP was 0.5–8.0%, and that of transient bacteremia was 27.00%^27^. Cholangitis after ERCP was one of the leading causes of postoperative acute death^28^. Therefore, early intervention for high-risk patients was necessary to reduce the incidence of cholangitis. If the risk of postoperative cholangitis was high before operation, we need to strictly control the operation time and extract bile for bacterial culture before radiography. A small and slow injection of contrast medium was appropriate to achieve the minimum amount of development, and reasonable selection of stent type and length can consider indwelling nasal bile duct or temporary biliary stent to play the role of biliary decompression. Nasobiliary duct can also be used for bile culture in postoperative cholangitis and bile duct irrigation to prevent the occurrence of postoperative biliary infection^29^.

To our knowledge, few studies have used machine learning-assisted models to predict the risk of cholangitis after ERCP stent implantation in Patients with MOJ. This study systematically studied the different applications of the prediction model combining the BAS algorithm and RF classification model. Machine learning classifiers were developed and evaluated to predict patients' cholangitis risk after ERCP, using indicators from 27 clinical sets as input features, and validated on independent test sets. Compared with the other 5 machine learning models. The results show that the prediction accuracy of the hyperparameter and feature selection optimized is the best by the BAS optimization algorithm (Table 3). In the case of large sample size, the model in this study will significantly reduce the prediction time of the model and save a lot of time cost.

From the univariant analysis in this study, we found that operation of time achieved an optimal AUROC of 0.83 (Fig. 2a). This result was in line with numerous research results and clinical experience^30,31^, indicating that operation of time was one of the most commonly used and widely verified clinical predictors of cholangitis after ERCP. Other variables presenting great potential include tumor type and obstruction position, indicating that these tumor-related elements may increase the risk of cholangitis. However, univariate analysis was not sufficient to analyze complex data or nonlinear data sets, and physicians cannot rely on a single indicator to make the diagnosis in clinical diagnosis.

Conversely, machine learning can work with complex and nonlinear data, often considering associations between variables and variable-target associations^32^. It can be seen from the results that the RF model optimized by the BAS algorithm has the best prediction accuracy (AUROC: 0.87). In addition, the AUROC of the three models was more significant than 0.75, and the prediction accuracy of BN and AB models was relatively poor (Fig. 2b and Table 3). To further advance, we used the BSA algorithm to optimize the hyperparameters and feature selection based on the RF model and SHAP feature importance analysis results. This way, we set up a clinical data set that better fits the model to reduce the risk of overfitting. As a result, the optimal AUROC of the model was 0.89 (Fig. 5).

Applying the RF model and SHAP method effectively calculated the essential input features. It provided the reference for the follow-up physicians' clinical diagnosis. In the prediction model of this study, the factors that were highly correlated with postoperative cholangitis were operation of time, DBIL (TBIL), albumin, blood glucose, WBC, HB, age, and obstruction location. Most patients with MOJ have increased total bilirubin, especially direct bilirubin. The study has shown that hyperbilirubinemia can stimulate cytotoxic reactions and reduce the defense ability of cells. The bilirubin was higher, and the risk of infection is greater^33^. Serum albumin and hemoglobin levels were essential indicators to measure the nutritional status of patients, and their levels directly reflected the patients' defense, immune, and self-recovery ability^34^. Hypoproteinemia and anemia were significant risk factors for postoperative infection, and the above nutritional status should be improved before surgery^35^. Blood glucose was related to cholangitis after ERCP. A high glucose environment in the body was conducive to the growth of biliary tract bacteria, and metabolic disorders in the body further affect immunity, resulting in difficult infection control^36,37^. The older the patient, the higher the incidence of postoperative complications. ERCP was safe and effective for elderly patients, but advanced age and high score of American Society of Aneshesiologists (ASA) physical status classification system will increase the risk of postoperative infection^38^. The typical physiological structure of the human body can have a particular defense function against the biliary system, such as sphincter can control the opening or closing of the pancreaticobiliary duct. However, in the ERCP operation, the extension of time was mainly due to tube difficulties, and repeated intubation resulting in damage to the sphincter, bile duct, pancreas, pancreatic duct, and other tissues. In this process, intestinal bacteria, epidermal bacteria and other bacteria were constantly brought into the bile duct, significantly increasing the risk of postoperative infection^39^. The prolonged operation time of ERCP can increase the amount of bleeding during the operation, prolong the time of anesthesia, and further lead to the decline of patients' immunity and increase the risk of postoperative infection. Therefore, high biliary obstruction increased the complexity of the procedure due to the presence of an obstruction in multiple branches, prolonging the operation time and leading to bacterial migration and postoperative complications of cholangitis. Hilar biliary obstruction has a higher risk of cholangitis after ERCP than distal biliary obstruction (OR [95% CI] 2.586 [2.066–2.743])^36^. Biliary stenosis exceeding 1.5 cm (OR [95% CI] 5.20 [2.23–12.16]; *P* = 0.000) was a risk factor for deep abdominal infection^40^. A malignant tumor was one of the fundamental causes of acute cholangitis, which may be caused by the decreased defense ability of patients with a malignant tumor and cytotoxic reaction caused by high bilirubin level, resulting in immune destruction^41^. However, the differences among different tumor types in this study were insignificant, and more sample sizes are needed to explore further the differences of cholangitis among different tumor types causing MOJ. However, patients' previous history of radiotherapy and chemotherapy and surgery had little influence, indicating that ERCP is a relatively effective and safe palliative conservative treatment measure.

Personalized diagnosis and treatment were clinical treatment goals in modern medicine^42^. Predictive models provided a personalized assessment of patient-specific clinical characteristics and were increasingly being incorporated into clinical practice in the medical field^43–45^. In the future, predictive models using machine learning methods can assist doctors in providing decision support to improve the accuracy of patient diagnosis and reduce clinical diagnostic errors^46^. The model developed in this study can be used to identify optimal classifiers quickly and applied to new datasets of various data, thus facilitating the transition from academic research to clinical practice.

While the study has many advantages, it also has some limitations. This was a retrospective study due to incomplete data and partial loss of follow-up data. In addition, these patients were all from the same hospital, which lacked the diversity of data. Therefore, the findings of this study still need to be validated in a large prospective multicenter study. The accuracy of the diagnostic model based on machine learning depends on the number of training samples. Large amounts of multidimensional medical data will be stored in the future, potentially improving the accuracy of machine learning-based classifiers.

## Conclusion

A hybrid artificial intelligence model combining BAS algorithm and RF classification model can successfully distinguish patients with cholangitis from patients with no-cholangitis based on a small number of routinely available laboratory indicators and may serve valuable adjunctive roles in the presence of diagnostic uncertainty or for expedited triage when blood test results and imaging are not readily available. We found that the ML model significantly improved the classification ability than univariate analysis and identified several clinically relevant variables that provide potential clinical indicators for clinical diagnosis. This study indicated that risk assessment based on machine learning could be used as an effective tool for early detection and decision-making in clinical practice for cholangitis.

### Supplementary Information

Supplementary Information.

## Data Availability

The data that support the findings of this study are available from the corresponding author upon reasonable request.

## Abbreviations

MOJ: Malignant obstructive jaundice
BMI: Body mass index
Child: Child–Pugh grade
DM: Diabetes mellitus
CH: Cholecystolithiasis
OP: Obstruction position
LO: Length of obstruction
HAS: History of abdominal surgery
PTCD: Percutaneous transhepatic cholangial drainage
ERCP: Endoscopic retrograde cholangiopancreatography
CHT: Chemo therapy
ST: Stent type
CHO: Cholangiocarcinoma
PC: Pancreatic cancer
DPC: Duodenal papillary carcinoma
AC: Ampulla carcinoma
MC: Metastatic cancer
WBC: White blood cell
HB: Hemoglobin
TBIL: Total bilirubin
DBIL: Direct bilirubin
Alb: Albumin
Glu: Blood glucose
OT: Obstruction of time
UA-ERCP: Used antibiotics before ERCP
OPT: Operation of time
