# Supplementary material for: Risk prediction of cholangitis after stent implantation based on machine learning
Source: Sci Rep. 2024 Jun 14;14:13715. doi: 10.1038/s41598-024-64734-w (PMC11178872; doi:10.1038/s41598-024-64734-w)
Supplement: Supplementary file 1 — Supplementary Information. [file 41598_2024_64734_MOESM1_ESM.docx]

**Risk prediction of cholangitis after stent implantation based on machine learning**

Rui Zhao^a^, Lin Gu^a^, Zhenzeng Ma^a^, Xiaojing Deng^a^, Dapeng Li^a^, Xiquan Ke^a^, Qizhi Wang^a^, Hailun Zheng^a^* , Yong Yang^b^*

^a^ The First Affiliated Hospital of Bengbu Medical University, Bengbu 233000, China;

^b^ School of Mechanical Engineering, Hefei University of Technology, Hefei 230009, China.

**Correspondence**

Hailun Zheng, The First Affiliated Hospital of Bengbu Medical University, Yanhuai Road, Bengbu 233000, China;

Yong Yang, School of Mechanical Engineering, Hefei University of Technology, Hefei 230009, China.

Email: 7369102@qq.com (H. Z); yongyang8686@163.com (Y.Y)

**1.1. Random forest**

Random forest (RF) model ^[^^[[1]](#endnote-1)]^ is a comprehensive classifier system composed of multiple random decision trees, whose output categories are determined according to the mode number of decision tree output category labels. It is one of the successful examples in integrated learning and has been widely applied in computer vision, pattern recognition, bioinformatics, and other fields. RF model can be understood as an improved method of random subspace decision forest ^[^^[[2]](#endnote-2)]^ method, random attribute feature selection ^[^^[[3]](#endnote-3)]^ method of Amit, and sampling aggregation algorithm ^[^^[[4]](#endnote-4)]^.

The RF model used the bagging technique to generate training data by resampling and replacing the original data set. This bagging feature enabled the RF algorithm to have better and higher prediction accuracy. The RF model can significantly improve the generalization ability and reduce the generalization error by using the optimal segmentation variables in the randomly sampled evidence feature subset in tree growth. RF model was also an effective method to evaluate the relative importance of each evidence feature and select the best feature in the prediction model ^[^^[[5]](#endnote-5)]^.

**1.2. Beetle Antennae Search Algorithm**

Beetle antennae search algorithm (BAS) was proposed in 2017 as a new biological heuristic intelligent global optimization algorithm ^[^^[[6]](#endnote-6),^ ^[[7]](#endnote-7)]^. Longicorn has two feelers longer than its body, which can sense the smell of food from far away and then foraging for food. Foraging behavior can be divided into moving behavior and turning behavior. First, the beetle used its antennae to explore the space around it, found the maximum direction for the food smell, turned in that direction, and then moved. For example, if the intensity of smell sensed by the right antenna were greater than that sensed by the left, the beetle would move to the right next step; otherwise, it would move to the left. After moving a certain distance, they used their feelers to explore the space around them, found the direction in which the food smelled the most, turned and moved again, and so on until they found the food. So, the smell of the food can be viewed as a fitness function in the optimization problem. This function has a different value at every point in space. The purpose of the beetle was to find the location of the food, which is the smelliest point in the whole world.

The specific process of transforming the foraging behavior of Longicorn beetles into an optimized search algorithm was as follows:

(1) Initialization parameters: For a K-dimensional optimal problem, the coordinates of the centroid were set as *x*, the coordinates of the left antenna search area were set as *x_l_*, and the coordinates of the right antenna search area were set as *x_r_*. The initial body length was *d_0_* between two antennas, and its value should be large enough to cover the appropriate search area for jumping out of the local minimum point at the beginning. The initial step size was set as *δ*, and its initial value should equal the search area, where *x*, *x_l_*, and *x_r_* are k-dimensional vectors.

(2) Randomly generate k-dimensional vector representing the vector pointing from the left whisker to the right whisker of Longicorn beetles, and normalize it into a unit vector:

$b=\frac{rand(k,l)}{\left\|rand(k,l)\right\|}$ （1）

where *rand ()* represents the random vector generating k dimensions.

The left antennae can be expressed as:

$x_{l}=x^{t}+\frac{dt\cdot b}{2}$ （2）

The right antennae can be expressed as:

$x_{r}=x^{t}-\frac{dt\cdot b}{2}$ （3）

where $x^{t}$ represents the position of the centroid of beetle antennae in the *t* iteration, and *dt* represents the distance between the two whiskers in the *t* iteration.

(3) Calculated the fitness values *f(x_l_)* and *f(x_r_)* of the two whiskers *x_l_* and *x_r_*, and judged the forward direction of longicorn beetles according to the size relationship of *f(x_l_)* and *f(x_r_)*:

$x^{t}=x^{t-1}-\delta^{t}\cdot b\cdot sign(f(x_{r})-f(x_{l}))$ （4）

Where *sign* is a sign function, and $\delta^{t}$ is the moving step of Longicorn beetle in the *t* iteration.

(4) Calculated the fitness value of beetle after moving, and updated the distance and step length of left and right antennas of beetle:

$\delta^{t}=A\cdot\delta^{t-1}$ （5）

$d^{t}=B\cdot d^{t-1}$ （6）

Where $d^{t}$ is the distance between the two antennas of beetles in the t-th iteration, and *A* and *B* are respectively the attenuation coefficient of the distance between the two antennas of beetles and the attenuation coefficient of step length, which is generally 0.95.

(5) Judged whether the end condition of iteration was met. The end iteration was met, otherwise steps (2), (3), (4) were repeated until the end condition is met.


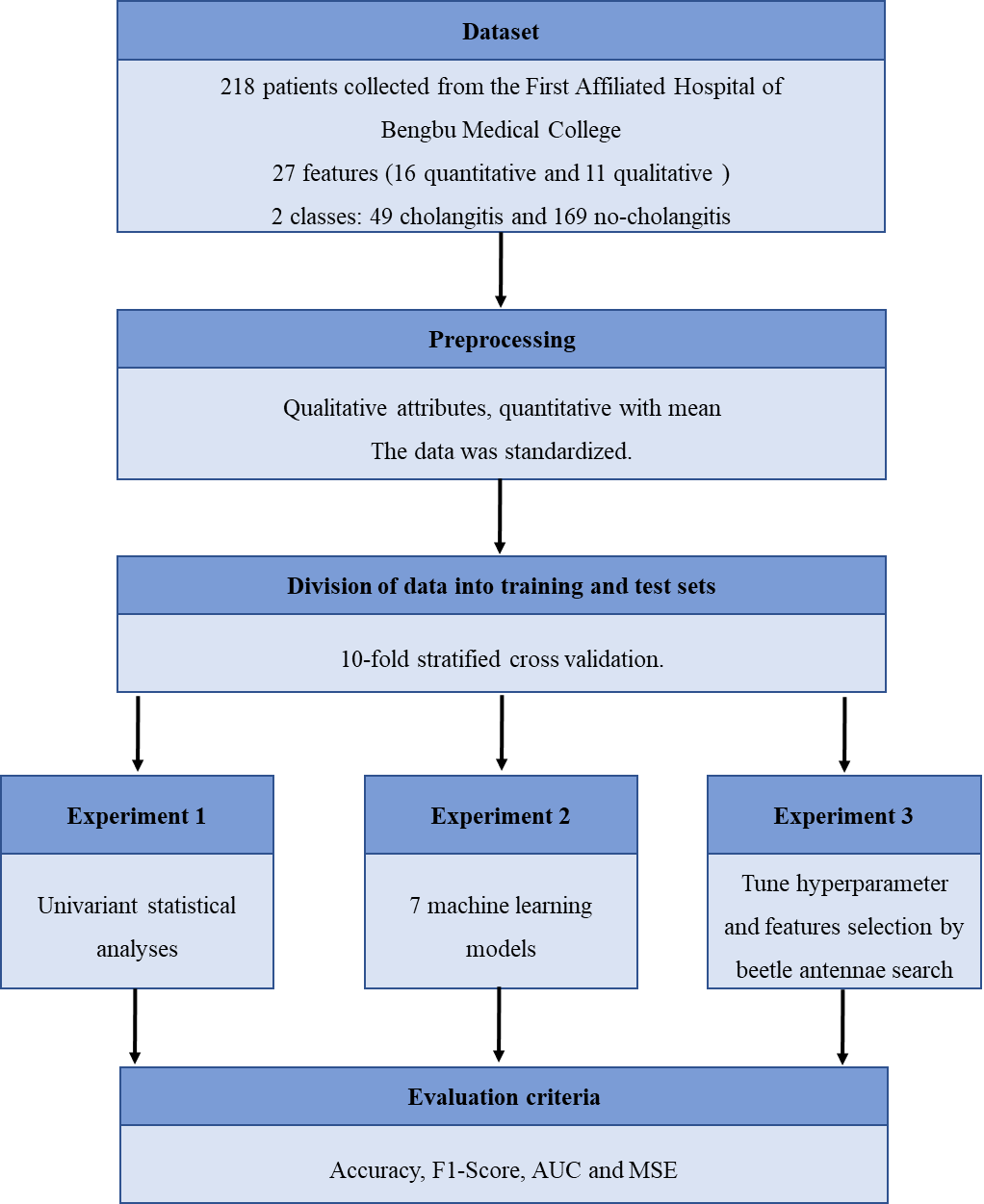


Fig. S1 Experiment schema

Table S1 the default and tuned parameters of the RF model.

| Parameter | Range | Default value | Tuned value |
| --- | --- | --- | --- |
| n_estimators | [10, 2000] | 100 | 452 |
| min_samples_split | [2, 10] | 2 | 9 |
| min_samples_leaf | [1, 10] | 1 | 2 |
| max_features | ["auto", "sqrt", "log2"] | "auto" | " sqrt " |
| max_depth | [1,100] | None | 70 |
| bootstrap | [True, False] | True | False |

Table S2 the model parameters of the RF model in selection features test.

| Parameter | Range | Value |
| --- | --- | --- |
| n_estimators | [10, 1000] | 452 |
| min_samples_split | [1, 10] | 9 |
| min_samples_leaf | [1, 10] | 2 |
| max_features | ["auto", "sqrt", "log2"] | " sqrt " |
| max_depth | [1,10] | 70 |
| bootstrap | [True, False] | False |
| Feature 1 | [0,1] |  |
| Feature … | [0,1] |  |
| Feature 15 | [0,1] |  |

1. **References**

   [] Breiman L. Random forests. *Mach Learn.* **45**, 5-32 (2001).

   <https://doi.org/10.1023/A:1018054314350>. [↑](#endnote-ref-1)
2. [] Breiman L, Friedman JH, Olshen RA, et al. Classification and regression trees. (CRC, 1984). [↑](#endnote-ref-2)
3. [] Amit Y, Blanchard G, Wilder K. Multiple randomized classifiers: MRC. Technical Report, Department of Statistics, University of Chicago,1999. [↑](#endnote-ref-3)
4. [] Breiman L. Bagging predictors. *Mach Learn.* **24**(2), 123-140 (1996). [↑](#endnote-ref-4)
5. [] Peters J, Baets BD, Verhoest NEC, et al. Random forests as a tool for ecohydrological distribution modelling. *Ecol Model.* **207**(2), 304-318 (2007).

   <https://doi.org/10.1016/j.ecolmodel.2007.05.011>. [↑](#endnote-ref-5)
6. [] Sun Y, Zhang J, Li G, et al. Optimized neural network using beetle antennae search for predicting the unconfined compressive strength of jet grouting coalcretes. *Int J. Numer Anal Met.* **43**(4), 801-813 (2019).

   <https://doi.org/10.1002/nag.2891>. [↑](#endnote-ref-6)
7. [] Jiang X, Li S. BAS: beetle antennae search algorithm for optimization problems. Arxiv Preprint Arxiv:1710.10724, **01**(1) (2017).

   <https://arxiv.org/abs/1710.10724>. [↑](#endnote-ref-7)
